# Family structure, socioeconomic status, and mental health in childhood

**DOI:** 10.1007/s00787-023-02329-y

**Published:** 2023-12-26

**Authors:** Laura Grüning Parache, Mandy Vogel, Christof Meigen, Wieland Kiess, Tanja Poulain

**Affiliations:** 1 LIFE Child, Hospital for Children and Adolescents, Leipzig University, Leipzig, Germany; 2 Department of Women and Child Health, Hospital for Children and Adolescents and Center for Pediatric Research (CPL), Leipzig University, Leipzig, Germany

**Keywords:** Family structure, Child mental health, Child health, Strengths and Difficulties Questionnaire, Socioeconomic status

## Abstract

The changing landscape of family structures over the last decades has led to a growing need to investigate its impact on children's well-being. This study examined differences in mental health among children from different family compositions and how these differences may be affected by familial socioeconomic status (SES). Data were collected within the LIFE Child study. Participants included 2828 children aged 3–17 years raised in traditional families, stepfamilies, or single-parent families. Mental health was measured using the Strengths and Difficulties Questionnaire (SDQ (behavioral strengths and difficulties)) and the KIDSCREEN-27 questionnaire (quality of life). Linear regression analyses were applied to examine associations between family structure, SES, and mental health outcomes. Children from single-parent families exhibited worse mental health outcomes than those from traditional families across all domains of the SDQ and the KIDSCREEN-27. Children from stepfamilies showed significantly higher Total Difficulties scores (*B* = 1.29 and 1.42), with 3- to 10-year-olds displaying higher scores in the Hyperactivity & Inattention (*B* = 0.61) and Peer Relationship Problems (*B* = 0.36) subscales, and 11- to 17-year-olds showing higher Conduct Problems (*B* = 0.31), Emotional Symptoms (*B* = 0.58), and a worse Parent Relationship scores (*B* = − 1.82) than children from traditional families (all *p* < 0.05). After controlling for SES, several associations between family structure and mental health lost significance, while others persisted, particularly among older children. To promote mental health in non-traditional families, interventions should address socioeconomic disparities while also investigating factors contributing to the direct impact of family structure on mental well-being.

***Trial registration*** The LIFE Child study is registered on clinicaltrials.gov (No. NCT02550236).

**Supplementary Information:**

The online version contains supplementary material available at 10.1007/s00787-023-02329-y.

## Introduction

Over the past half century, the arrangement and composition of families, which is referred to as family structure, has experienced significant changes in Western societies [1–4]. Increased rates of partnership instability, such as divorces and separations, have led to a shift in the normative “traditional family”, referring to a family structure in which a child lives with both of their biological parents, and led to a rising number of non-traditional (also referred to as alternative) family constellations [5]. These include single-parent families, where a child lives with one biological parent; stepfamilies, in which one or both partners in a couple have children from a previous relationship; as well as other less common forms of family composition, such as adoptive and foster families, grandparent-headed families and co-parenting arrangements. The emergence of diverse family structures where children are being raised is accompanied by a growing need to investigate the impact of these structures on children's well-being [6, 7].

Although there are no recent official statistics on the exact number of stepfamilies, single-parent families, and traditional families in Germany, the first wave of the “Generations and Gender Survey” (GGS) conducted in 2005 revealed that traditional families accounted for 70.8% of households with minor children, while single-parent families and stepfamilies made up 15.2% and 13.5%, respectively. In Europe as a whole, the average distribution of family structures differed somewhat, with approximately 80.0% classified as traditional families, 11.5% as single-parent families, and 7.9% as stepparent families [8].

As research has shown, familial composition can considerably influence children’s health and behavior. Living with a single parent or in stepfamilies is related to less physical activity, reduced participation in sports and increased screen time [9, 10]. Additionally, children living in these family arrangements tend to have poorer academic and health outcomes, including worse mental health, than children living with both parents in the same household [11–13]. For example, children raised in non-traditional families have been found to experience more internalizing and externalizing problem behaviors, more socio-emotional development deficiencies and are nearly twice as likely to experience mental disorders such as anxiety disorders, major depressive disorders, attention deficit hyperactivity disorder, and conduct disorders compared to children who grow up in traditional families [14, 15].

While there is ample empirical evidence that these correlations exist, the mechanisms behind these relationships remain controversial. Many factors, such as selection processes or the experience of critical life situations in the form of family breakdown, might play a role [16–18]. It is also important to note that shifts in familial arrangements have not affected all demographic groups equally [19]. In fact, distinctions can be seen based on factors such as income, profession, and educational level, with non-traditional family structures being more prevalent in households of lower socioeconomic status [7, 19, 20]. Specifically, the rise in single-mother households is particularly prevalent among those of lower socioeconomic status [21].

These trends contribute to an increasing stratification in family systems between the advantaged and the disadvantaged [7, 20]. As a result, inequalities in child-rearing resources have increased, emphasizing the importance of comprehending how varying levels of resources and socioeconomic status relate to different family structures and affect the health and well-being of children and adolescents [6, 19].

In 2019, Poulain et al. conducted a study on the relationship between family socioeconomic status (SES) and children’s health. Results showed that higher SES was associated with better health and a higher quality of life [22]. Children from higher social classes showed fewer behavioral difficulties, had healthier lifestyles and a lower body mass index, consumed less nicotine, and spent less time watching television. They also engaged in more physical activity, had better academic outcomes, and experienced fewer critical life events compared to children from lower social classes [22]. Results from the “Health Behavior in School-aged Children” study in Spain showed that adolescents with a higher perceived SES self-reported better health and fewer psychosomatic complaints [23]. In addition, high family income was found to be a protective factor for children’s emotional well-being [24]. These findings are consistent with results of the BELLA Study in Germany ("BELLA—Psychosocial Health of Children and Adolescents in Germany") and similar studies conducted in countries such as the UK and Australia [23, 25–27]. The above-mentioned findings emphasize the potential significance of familial socioeconomic status in relation to our research questions.

The primary objectives of this study were twofold: first, we aimed to gain insights into the interplay between distinct family structures and childhood mental health. This encompassed an examination of both internalizing (Emotional Symptoms and Peer Relationship Problems) and externalizing (Hyperactivity & Inattention and Conduct Problems) problem behaviors as well as the quality of life, considering various dimensions including Physical Well-Being, Psychological Well-Being, Parent Relation & Home Life, Social Support & Peers, and School Environment. A second aim was to investigate the potentially moderating role of socioeconomic status (SES), as well as age and sex, in shaping the relationship between family structure and child mental health. In recent years, research has delved into these associations. However, the majority of these studies have primarily focused on children raised by single parents. In Germany, previous research projects have often employed simplified approaches, either examining dichotomized mental health scores or utilizing composite scores, rather than exploring various mental health and quality of life indicators. Here, we offer a more detailed understanding of various facets of children's well-being. Formulating our hypotheses, we predicted that children in non-traditional family structures would likely display lower mental health scores in contrast to those in traditional family settings. In particular, we expected that children raised by single parents would face the most notable difficulties in this aspect. Furthermore, we anticipated that these observed effects would diminish when considering the family’s socioeconomic status.

## Methods

### Study design and population

The present study was conducted as part of the LIFE Child study, a cohort study initiated in 2011 and performed at the LIFE Research Centre for Civilization Diseases at the University of Leipzig, Germany. It aims to gain insights into the effects of environmental, metabolic and genetic factors on the development of healthy children and adolescents [28]. During annual visits to the study outpatient clinic, participants undergo various medical, psychological, and sociodemographic examinations [29]. The LIFE Child study is funded by the European Union, the European Social Fund (ESF), the European Regional Development Fund (ERDF), and the Free State of Saxony.

The data for the present study were collected between 2011 and 2022 from 3980 participants residing in Leipzig and its surroundings, with a total number of 14134 visits. We excluded children lacking information on sociodemographic parameters and children with incomplete questionnaires on internalizing and externalizing behavioral difficulties and quality of life, as well as participants where the family structure was not a traditional family, stepfamily, or single-parent family, since they constituted only 0.46% of the sample. Furthermore, to avoid confounding by multiple visits and family relationships within the sample, we only considered the last visit by each child and only the youngest child of each family. The final sample comprised 2828 children (52.3% males) aged between 3 and 17 years old, with age rounded to the nearest half year (2.5 to 17.5 years, mean = 10.4 years, SD = 4.45). Analyses were conducted separately for two age groups: 3- to 10-year-olds, where the questionnaires were completed by the parents, and 11- to 17-year-olds, who self-reported on their behavior and quality of life. Children at that age are well able to understand the content of questionnaires and to complete them accurately.

The LIFE Child study was approved by the Ethical Committee (Institutional Review Board) of the Medical Faculty, University of Leipzig (477/19-ek). The study protocol was designed in accordance with the Declaration of Helsinki.

### Measures

#### Family structure

The family structure of the study population was assessed using a questionnaire on household and family living conditions developed by LIFE Child. This questionnaire is based on the family well-being scale of the international HBSC study (Health Behaviour in School-aged Children) [30] and in accordance with standards and recommendations of the national KiGGS-study (German Health Interview and Examination Survey for Children) [39]. Parents were asked the question “Who does your child live with most of the time?”. The classification of family structure includes traditional families, in which the child resides with both biological parents, regardless of their marital status; stepfamilies, where the child lives with one biological parent and her/his partner; and one-parent families, where the child lives with only one biological parent. For children living in shared custody arrangements (spending half their time with each parent), the responding parent was asked to choose one of the latter two constellations.

#### Mental health and quality of life

Behavioral strengths and difficulties were evaluated using the self and parent versions of the German Strengths and Difficulties Questionnaire (SDQ). The SDQ is a widely used and psychometrically robust brief questionnaire designed to identify behavioral difficulties and prosocial resources in children of nursery and school age [32, 33]. The assessment is based on five scales: Emotional Symptoms, Behavioral Problems, Hyperactivity & Inattention, Peer Relationship Problems, and Prosocial Behavior. Each scale consists of five questions answered on a three-point Likert scale, resulting in scale values ranging from 0 to 10, with higher scores indicating more problems or strengths. The Prosocial Behavior scale reflects a behavioral strength, while all other scales reflect internalizing (in the case of Emotional Symptoms and Peer Relationship Problems) or externalizing (in the case of Hyperactivity & Inattention and Conduct Problems) behavioral difficulties. The scales indicating problems are added together to establish the Total Difficulties score. Cronbach´s alpha reliability coefficients ranged between 0.58 (Conduct Problems) and 0.8 (Hyperactivity & Inattention) in the parent-report version and between 0.53 (Conduct Problems) and 0.73 (Hyperactivity & Inattention) in the self-report version of the questionnaires.

Health-related quality of life (HRQOL) was assessed using the KIDSCREEN-27 Questionnaire. It is a standardized measure with 5 Rasch-scaled dimensions, namely Physical Well-Being, Psychological Well-Being, Parent Relationship & Home Life, Peers & Social Support, and School Environment. All questions are answered on a five-point Likert scale, resulting in scores where higher values reflect higher HRQOL. It is a widely used measure in European countries [34]. The questionnaire can only be completed by children between 8 and 18 years of age; therefore, we only analyzed quality of life in the age group of 11- to 17-year-olds. Cronbach´s alpha reliability coefficients in this group ranged from 0.79 (Parent Relation & Home Life) to 0.87 (Psychological Well-Being).

#### Socioeconomic status

The study participants’ socioeconomic status (SES) was determined through a questionnaire, originally designed for the German Health Interview and Examination Survey for Children and Adolescents (KiGGS) [35]. The questionnaire assesses parents’ education, occupation, and income. Family income is converted into equivalized disposable income by taking into consideration the number of adults and children living in the same household. Education includes both formal schooling and vocational training. Each indicator is given a score between 1 and 7. These scores are then combined into a composite score ranging from 3 to 21. The higher the index score, the higher the SES. The index is used to classify families into lower, middle, or higher SES. In a representative sample, 20% of the sample should belong to the lower SES, 60% to the middle SES, and 20% to the higher SES [35].

### Statistical analyses

All analyses were conducted using R version 4.2.1 (R Foundation for Statistical Computing, Vienna, Austria) [36]. Stratification by age groups (3- to 10-year-olds, 11- to 17-year-olds) was performed. To assess associations between socioeconomic status score (dependent variable) and family structure (independent variable), we performed linear regression models. Thereafter, linear regression models were fitted to examine the associations between behavioral difficulties and quality of life (dependent variables) and family structure (reference category: traditional family) and socioeconomic status (independent variables). First, the independent variables were included in separate models. In a second step, they were included simultaneously in a model to assess whether the associations were independent. All associations were adjusted for child age and sex.

Finally, a moderation analysis was performed to examine the interactions between family structure and potential moderating variables (sex, age, and socioeconomic status) in association with mental health. Interactions were considered statistically significant at a threshold of *p* < 0.05 and were retained in the final models only if they did not result in multicollinearity (i.e., VIF < 5).

The significance level was set to α = 0.05. P-values were adjusted for multiple comparisons using the Benjamini–Hochberg procedure [37]. Unlike the Bonferroni method, which is highly conservative and primarily focuses on minimizing Type I errors, the BH procedure strikes a balance between controlling for Type I errors and maximizing statistical power [38].

## Results

### Sample characteristics

The participants’ characteristics are summarized in Table 1. The final sample incorporated 2828 healthy children and adolescents, 1522 children aged 3–10 years, and 1306 children aged 11–17 years. The sample's family structure was primarily traditional families, followed by one-parent families and stepfamilies. The participants’ socioeconomic status was predominantly medium, followed by high and low SES. Younger children (aged 3–10 years) had the highest scores in the scales reflecting externalizing behavioral difficulties (Hyperactivity & Inattention, and Conduct Problems), whereas older children had the highest scores in scales indicating internalizing behavioral difficulties (Emotional Problems and Peer Relationship Problems).

**Table 1:** Sample characteristics

	Age group			
	3- to 10-year-olds (*n* = 1522)		11- to 17-year-olds (*n* = 1306)	
	N	%	N	%
Sex				
Male	824	54.1	655	50.2
Female	698	45.9	651	49.8
Age (years, mean ± SD)	6.82	± 2.46	14.6	± 1.93
Family structure				
Traditional family	1143	75.1	820	62.8
One-parent family	241	15.8	335	25.7
Mother	229	15.1	307	23.5
Father	12	0.79	28	2.14
Stepfamily	138	9.07	151	11.6
Mother and partner	130	8.54	134	10.3
Father and partner	8	0.53	17	1.30
Socioeconomic status				
High	578	38.0	368	28.2
Medium	820	53.9	782	59.9
Low	124	8.15	156	11.9
SDQ score (mean ± SD)				
Total Difficulties	9.03	± 5.36	10.0	± 5.33
Conduct Problems	2.10	± 1.60	1.64	± 1.43
Emotional Symptoms	1.84	± 1.86	2.54	± 2.20
Hyperactivity & Inattention	3.77	± 2.40	3.52	± 2.20
Peer Relationship Problems	1.31	± 1.57	2.30	± 1.77
Prosocial Behavior	7.87	± 1.68	7.83	± 1.80
KIDSCREEN-27 scores (mean ± SD)				
Physical Well-Being			49.10	± 9.32
Psychological Well-Being			49.59	± 10.0
Parent Relation & Home Life			53.69	± 9.75
Social Support & Peers			51.75	± 10.26
School Environment			51.41	± 9.34

### Associations between family structure and SES

**Fig. 1:**
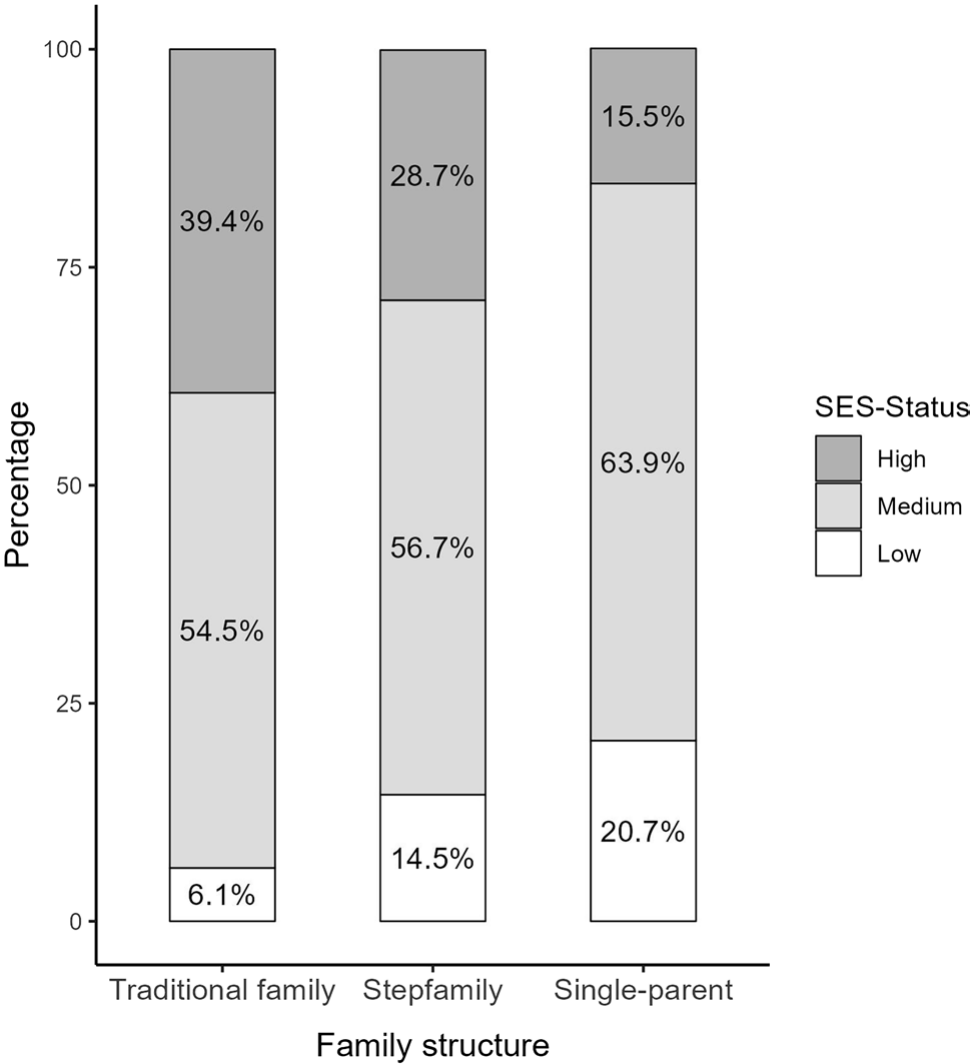
Distribution of SES by family structure

SES was highest in traditional families (3- to 10-year-olds, mean = 15.10; and 11- to 17-year-olds, mean = 14.17). The analyses revealed a significantly lower SES in single-parent-families (*B* = − 3.18, *p* < 0.001 and *B* = − 2.38, *p* < 0.001, respectively) and in stepfamilies (*B* = − 1.42, *p* < 0.001 and *B* = − 1.06, *p* = 0.003, respectively). The differences in the distribution of traditional families, stepfamilies and single-parent families with high, medium, and low socioeconomic status are shown in Fig. 1. The share of high-SES families was highest in traditional families, while the amount of low SES families was highest in single-parent families.

### Associations of family structure and SES with mental health

A higher SES was associated with significantly fewer behavioral difficulties and significantly higher quality of life in all domains of the SDQ and KIDSCREEN-27 (see Tables 2 and 3). Except for the relationship between SES and Prosocial Behavior in the younger age group, all these associations remained significant after adjusting for family structure.

**Table 2:** Associations between family structure and SES (independent variables) and behavioral strengths and difficulties (dependent variables) in 3- to 10-year-olds, results of multiple regression analyses

	Dependent variables, SDQ					
	Total Difficulties score	Conduct Problems	Hyperactivity & Inattention	Emotional Symptoms	Peer Relationship Problems	Prosocial Behaviour
Separate models for independent variables						
FS^1^, stepfamily	1.42**	0.19	0.61**	0.27	0.36*	0.00
FS^1^, single-parent	2.07***	0.43***	0.69***	0.65***	0.31*	-0.25
(Partial R^2^)	0.02	< 0.01	0.01	0.02	< 0.01	< 0.01
SES	− 0.37***	− 0.07***	− 0.14***	− 0.09***	− 0.08***	0.03***
(Partial R^2^)	0.07	0.03	0.05	0.03	0.04	< 0.01
Independent variables included simultaneously in one model						
FS^1^, stepfamily	0.94	0.10	0.43	0.16	0.25	0.03
FS^1^, single-parent	0.99*	0.23	0.29	0.40**	0.07	**− **0.17
(Partial R^2^)	< 0.01	< 0.01	< 0.01	< 0.01	< 0.01	< 0.01
SES	− 0.34***	− 0.06***	− 0.13***	− 0.08***	− 0.08***	0.02
(Partial R^2^)	0.05	0.02	0.04	0.02	0.03	< 0.01

^*^p < 0.05, **p < 0.01, ***p < 0.001; significant associations are highlighted in bold

^1^ FS: Family structure (reference = traditional family); all associations adjusted for age and sex

**Table 3:** Associations between family structure and SES (independent variables) and behavioral strengths and difficulties and quality of life indicators (dependent variables) in 11- to 17-year-olds, results of multiple regression analyses

	Dependent variables, SDQ					
	Total Difficulties score	Conduct Problems	Hyperactivity & Inattention	Emotional Symptoms	Peer Relationship Problems	Prosocial Behaviour
Separate models for independent variables						
FS^1^, stepfamily	1.29*	0.31*	0.29	0.58**	0.11	-0.36*
FS^1^, single-parent	1.77***	0.31**	0.60***	0.44**	0.42***	-0.17
(Partial R^2^)	0.02	0.01	0.01	0.01	0.01	< 0.01
SES	− 0.24***	− 0.05***	− 0.06***	− 0.05**	− 0.09***	0.05***
(Partial R^2^)	0.03	0.01	0.01	< 0.01	0.04	< 0.01
Independent variables included simultaneously in one model						
FS^1^, stepfamily	1.08*	0.27*	0.24	0.54**	0.02	**− **0.31
FS^1^, single-parent	1.29***	0.22*	0.50**	0.34*	0.22	**− **0.06
(Partial R^2^)	0.01	< 0.01	< 0.01	< 0.01	< 0.01	< 0.01
SES	− 0.20***	− 0.04***	− 0.04*	− 0.04*	− 0.08***	0.04**
(Partial R^2^)	0.02	< 0.01	< 0.01	< 0.01	0.03	< 0.01

	Dependent variables, KIDSCREEN-27				
	Physical Well-Being	Psychological Well-Being	Parent Relation & Home Life	Social Support & Peers	School Environment
Separate models for independent variables					
FS^1^, stepfamily	0.55	− 1.37	− 1.82*	1.07	− 1.00
FS^1^, single-parent	− 2.51***	− 2.24***	− 2.32***	− 1.69*	− 1.33*
(Partial R^2^)	0.02	0.01	0.01	< 0.01	< 0.01
SES	0.40***	0.27***	0.45***	0.22**	0.26***
(Partial R^2^)	0.03	0.01	0.03	< 0.01	0.01
Independent variables included simultaneously in one model					
FS^1^, stepfamily	0.93	− 1.13	− 1.39	1.28	− 0.75
FS^1^, single-parent	− 1.66*	− 1.72*	− 1.35	− 1.24	− 0.77
(Partial R^2^)	< 0.01	< 0.01	< 0.01	< 0.01	< 0.01
SES	0.36***	0.22**	0.41***	0.19*	0.24**
(Partial R^2^)	0.02	< 0.01	0.02	< 0.01	< 0.01

^*^p < 0.05, **p < 0.01, ***p < 0.001; significant associations are highlighted in bold

^1^ FS: Family structure (reference = traditional family); all associations adjusted for age and sex

Regarding family structure, regression analyses revealed that children aged 3–10 years from stepfamilies had significantly higher Total Difficulties scores as well as higher scores on the Hyperactivity & Inattention, and Peer Relationship Problems scales than children from traditional families. Children from single-parent families had significantly higher scores on all problem scales of the SDQ, indicating more internalizing and externalizing behavioral difficulties (see Table 2).

Similar results were observed for 11- to 17-year-olds, as children from stepfamilies had significantly higher Total Difficulties scores and higher scores on the Conduct Problems and Emotional Symptoms scales than children from traditional families. Participants from single-parent families again had higher scores on all problem scales of the SDQ (see Table 3). Furthermore, children aged 11–17 years from single-parent families showed significantly lower scores in all scales constituting the KIDSCREEN-27 (see Table 3), indicating a lower quality of life than children from traditional families. Children from stepfamilies had significantly lower scores on the Parent Relation & Home Life scale than children in traditional families.

After including the independent variables (family structure and SES) simultaneously in linear regression models, the results showed that several associations between family structure and mental health lost significance (see Tables 2 and 3). However, some significant differences in mental health outcomes depending on family structure remained. Children aged 3–10 years from single-parent families still had higher Total Difficulties scores and Emotional Symptoms scores compared to children from traditional families.

In older children (11- to 17-year-olds), most associations remained significant (see Table 3). However, the associations between stepfamily status and Prosocial Behavior and Parent Relation & Home Life as well as the associations between single-parent family status and Peer Relationship Problems, Parent Relation & Home Life, Social Support, and School Environment lost statistical significance after adjusting for SES.

### Moderator analyses

The moderator analysis revealed two significant interactions between family structure and child sex in 11- to 17-year-olds. The first interaction indicated that the difference between child Prosocial Behavior in single-parent families (lower Prosocial Behavior) versus traditional families (higher Prosocial Behavior) was only significant for boys (*B* = − 0.33, *p* = 0.047), but not for girls (*B* = 0.19, *p* = 0.235). The second interaction showed that the difference between stepfamilies and traditional families regarding Physical Well-Being was again only significant for boys (*B* = 3.48, *p* = 0.003) but not for girls (*B* = − 1.38, *p* = 0.207). Surprisingly, the significant association in boys indicated higher Physical Well-Being in boys from stepfamilies than in boys from traditional families.

The analyses revealed no interactions in the 3- to 10-year-old group. Also, neither age nor SES moderated the associations between family structure and child mental health and quality of life.

## Discussion

The results of this study suggest that family structure is an important factor influencing children's well-being. Children from single-parent families displayed more internalizing and externalizing behavioral difficulties as well as a lower quality of life than their counterparts from traditional families. Children from stepfamilies showed more behavioral difficulties and reported a worse relationship with their parents compared to children from traditional families. However, they did not differ from children from traditional families in other quality of life domains. These findings are in line with our hypotheses and the results of previous studies showing that children from single-parent families are a particularly vulnerable population [39], possibly due to various factors, including financial strain, parental stress, and inadequate parenting resources [19, 25]. The study's finding that children from stepfamilies also had higher levels of behavioral difficulties compared to those from traditional families may be explained by the challenges associated with blending families [40, 41]. It is possible that the absence of one parent or the presence of a stepparent may create a more challenging family environment, leading to increased stress and emotional difficulties for children [42].

In addition to family structure, the study findings also emphasize the significance of familial socioeconomic status (SES) for children's mental health outcomes. Specifically, higher SES was associated with significantly fewer behavioral difficulties and higher quality of life in all the assessed domains, accenting the beneficial impact of economic resources on children's well-being. This finding is consistent with previous research that has linked socioeconomic disadvantage to increased risk of mental health problems in children [22–24].

Importantly, the study also revealed that family structure and SES are interrelated, with alternative families (single-parent and stepfamilies) showing significantly lower SES than traditional families. It is possible that the financial strain associated with non-traditional family structures may create an additional burden for households, creating a more challenging family environment and increased mental health problems for children.

After including both family structure and SES in the regression models, several associations between family structure and mental health outcomes lost significance, while associations between SES and mental health did not. This suggests that SES may have a stronger effect on mental health and may partially account for the relationship between family structure and mental health outcomes in children. This emphasizes the significance of economic resources for children’s mental well-being. However, some significant differences in mental health outcomes depending on family structure remained, especially in 11- to 17-year-olds, suggesting that family structure may also have a direct effect on children's mental health, which increases and accumulates over time and therefore is stronger in older than in younger children. Nevertheless, it is worth noting that the variations in the observed relationships between younger (ages 3–10) and older (ages 11–17) children might also be attributed to the differences in data collection methods. Parental perceptions and interpretations of a child´s behavior and well-being might introduce some level of bias. Older children, self-reporting independently, might provide more direct insight into their own experiences.

The results of the moderation analyses suggest that the relationship between family structure and child outcomes may vary depending on the child's sex. Boys may be particularly vulnerable to the negative effects of single-parent families on Prosocial Behavior but may benefit more from being in a stepfamily in terms of Physical Well-Being. These findings were only made in the 11- to 17-years-old group. One possible reason for this finding might be the presence of an additional (male) role model or stepsiblings. Positive relationships with other adults and children within stepfamilies might encourage physical activity and healthy habits, indirectly contributing to better physical well-being. Unfortunately, we did not assess whether there were additional children in the stepfamilies.

Our analysis did not uncover any significant moderating effects of age or, notably, socioeconomic status on the relationship between family structure and child outcomes. This suggests that the effects of family structure on child outcomes remain consistent across different levels of socioeconomic status. Although family structure and SES are related factors that can both influence child mental health, it's important to note that their effects are not necessarily additive. This is because some of the effects of family structure may already be explained by socioeconomic status. In the same way, high socioeconomic status does not necessarily mitigate the effects of "unfavorable" family constellations, and vice versa. Overall, the results described above suggest that the impact of family structure on mental health outcomes may be partially explained by differences in SES between families. This finding underlines the importance of combating social inequality and poverty within society (e.g., by establishing inexpensive/free all-day programming, including food and extracurricular activities such as sports or music) to improve children’s health and development. However, the direct impact of family structure on children's well-being also points to the need to further investigate the underlying factors contributing to this association, such as quality and quantity of relationships within the family. Qualitative research may provide a more subtle understanding of these factors. This, in turn, can facilitate the development of nuanced, flexible, and comprehensive interventions that take into account the unique needs and circumstances of families and empower them to discover individually tailored solutions through an asset-based approach.

## Strengths and limitations

The comprehensive approach of this study, examining various facets of children's well-being using the Strengths and Difficulties Questionnaire and the KIDSCREEN-27 in their entirety, represents the strength of this research. This provides a more detailed understanding of the dynamics at play. While recent years have seen increased exploration of these associations, the majority of studies have primarily focused on children raised by single parents. In Germany, prior research often employed simplified approaches, limiting the examination to dichotomized mental health scores or composite scores, rather than exploring multiple mental health and quality of life indicators. This study addresses these gaps by offering a more nuanced understanding of children's well-being within the context of family structure and SES.

It is essential to recognize some limitations of this study. First, the overrepresentation of families from higher socioeconomic backgrounds relative to the German population limits the generalizability of the findings to families from lower socioeconomic positions. Another limitation of the study is that it did not include families with shared custody arrangements (co-parenting). This presents a drawback, as shared custody is becoming increasingly common among non-traditional families, and the dynamics in these families may differ from those within other alternative family structures. Additionally, the study did not assess the quality of family relationships, which may also influence mental health outcomes in children. Finally, the focus on only one visit per child, i.e., the cross-sectional nature of the study design limits our ability to establish causality between family structure, SES, and mental health outcomes. Consequently, longitudinal studies are needed to provide a more in-depth understanding of the developmental trajectories of children from different family structures and socioeconomic positions, allowing for more targeted and effective interventions.

## Conclusion

This study sheds light on the interplay between family structure, socioeconomic status, and children's mental health and quality of life outcomes. While some effects of family structure may be explained by socioeconomic status, the direct impact of family dynamics remains significant, especially in older children. These findings underscore the need for interventions that address both socioeconomic disparities and the specific challenges faced by non-traditional families, particularly single-parent families. Further qualitative research can provide deeper insights into the underlying factors at play, guiding the development of customized, asset-based solutions for families.

### Supplementary Information

Below is the link to the electronic supplementary material.

Supplementary file1 (DOCX 37 KB)

## Data Availability

Data collected in the LIFE Child study are not publicly available, as the publication of data is not covered by the informed consent provided by study participants. Since data sets contain potentially sensitive information, all researchers seeking to access the data are required to sign project agreement. Researchers interested in accessing and analyzing data from the LIFE Child study may contact the data use and access committee (forschungsdaten@medizin.uni-leipzig.de).
