# Supplementary material for: Family structure, socioeconomic status, and mental health in childhood
Source: Eur Child Adolesc Psychiatry. 2023 Dec 26;33(7):2377–86. doi: 10.1007/s00787-023-02329-y (PMC11255005; doi:10.1007/s00787-023-02329-y)
Supplement: Supplementary file 1 — Supplementary file1 (DOCX 37 KB) [file 787_2023_2329_MOESM1_ESM.docx]

**SUPPLEMENT**

**Table 4:** Mean values and standard deviations, **3- to 10-year-olds**, n = 1522

|  | **Family structure** | | |
| --- | --- | --- | --- |
|  | Traditional family  (n = 1143) | Stepfamily  (n = 138) | Single-parent family  (n = 241) |
| **Questionnaires** | Mean (SD) | Mean (SD) | Mean (SD) |
| SDQ score |  |  |  |
| -Total Difficulties score | 8.60 (5.02) | 9.85 (6.38) | 10.6 (5.93) |
| -Conduct Problems | 2.04 (1.56) | 2.10 (1.67) | 2.41 (1.74) |
| -Emotional Symptoms | 1.70 (1.77) | 2.09 (2.07) | 2.40 (2.01) |
| -Hyperactivity & Inattention | 3.63 (2.36) | 4.09 (2.60) | 4.27 (2.41) |
| -Peer Relationship Problems | 1.23 (1.49) | 1.57 (1.74) | 1.54 (1.77) |
| -Prosocial Behavior | 7.90 (1.66) | 7.99 (1.67) | 7.66 (1.79) |
|  |  |  |  |
| SES score |  |  |  |
| -Total score | 15.1 (3.50) | 13.6 (4.00) | 11.9 (3.65) |
| -Education | 5.55 (1.43) | 5.06 (1.52) | 4.70 (1.51) |
| -Occupation | 4.44 (1.18) | 4.25 (1.30) | 3.79 (1.17) |
| -Income | 5.16 (1.74) | 4.31 (2.11) | 3.50 (1.90) |
|  |  |  |  |
|  | **Socioeconomic status** | | |
|  | High (n = 578) | Medium (n = 820) | Low (n = 124) |
| **Questionnaires** | Mean (SD) | Mean (SD) | Mean (SD) |
| SDQ score |  |  |  |
| -Total Difficulties score | 7.87 (4.83) | 9.35 (5.36) | 12.3 (6.06) |
| -Conduct Problems | 1.89 (1.50) | 2.17 (1.62) | 2.65 (1.80) |
| -Emotional Symptoms | 1.58 (1.74) | 1.88 (1.85) | 2.85 (2.07) |
| -Hyperactivity & Inattention | 3.29 (2.28) | 3.96 (2.40) | 4.76 (2.48) |
| -Peer Relationship Problems | 1.11 (1.42) | 1.33 (1.56) | 2.09 (2.01) |
| -Prosocial Behavior | 7.96 (1.70) | 7.83 (1.65) | 7.69 (1.82) |

|  |  | **Family structure** |  |
| --- | --- | --- | --- |
|  | Traditional family  (n = 820) | Stepfamily  (n = 151) | Single-parent family  (n = 335) |
|  |  |  |  |
| **Questionnaires** | Mean (SD) | Mean (SD) | Mean (SD) |
| SDQ score |  |  |  |
| -Total Difficulties score | 9.34 (5.15) | 10.7 (5.41) | 11.3 (5.47) |
| -Conduct Problems | 1.52 (1.38) | 1.81 (1.48) | 1.87 (1.50) |
| -Emotional Symptoms | 2.34 (2.11) | 3.01 (2.39) | 2.81 (2.28) |
| -Hyperactivity & Inattention | 3.32 (2.16) | 3.58 (2.21) | 3.98 (2.21) |
| -Peer Relationship Problems | 2.17 (1.70) | 2.29 (1.64) | 2.62 (1.96) |
| -Prosocial Behavior | 7.91 (1.76) | 7.58 (1.82) | 7.73 (1.89) |
|  |  |  |  |
| KIDSCREEN-27 score |  |  |  |
| -Physical Well-Being | 49.7 (9.25) | 50.0 (9.74) | 47.2 (9.07) |
| -Psychological Well-Being | 50.3 (9.73) | 48.7 (11.2) | 48.1 (9.92) |
| -Parent Relation & Home Life | 54.5 (9.53) | 52.6 (9.80) | 52.1 (10.0) |
| -Social Support & Peers | 52.1 (10.0) | 53.1 (9.17) | 50.4 (11.1) |
| -School Environment | 51.8 (8.88) | 50.7 (9.16) | 50.7 (10.4) |
|  |  |  |  |
| SES score |  |  |  |
| -Total score | 14.2 (3.56) | 13.1 (3.87) | 11.8 (3.74) |
| -Education | 5.05 (1.45) | 4.65 (1.41) | 4.63 (1.50) |
| -Occupation | 4.24 (1.19) | 4.01 (1.27) | 3.79 (1.08) |
| -Income | 4.92 (1.77) | 4.48 (1.97) | 3.52 (1.89) |
|  | **Socioeconomic status** | | |
|  | High (n = 368) | Medium (n = 782) | Low (n = 156) |
|  |  |  |  |
| **Questionnaires** | Mean (SD) | Mean (SD) | Mean (SD) |
|  |  |  |  |
| SDQ score |  |  |  |
| -Total Difficulties score | 9.00 (4.92) | 10.1 (5.36) | 11.6 (5.68) |
| -Conduct Problems | 1.51 (1.37) | 1.62 (1.42) | 2.08 (1.58) |
| -Emotional Symptoms | 2.24 (2.10) | 2.65 (2.22) | 2.70 (2.27) |
| -Hyperactivity & Inattention | 3.36 (2.23) | 3.51 (2.19) | 3.92 (2.11) |
| -Peer Relationship Problems | 1.89 (1.57) | 2.36 (1.73) | 2.94 (2.16) |
| -Prosocial Behavior | 8.02 (1.75) | 7.83 (1.76) | 7.33 (2.02) |
| KIDSCREEN-27 score |  |  |  |
| -Physical Well-Being | 50.9 (9.30) | 48.7 (9.09) | 46.9 (9.85) |
| -Psychological Well-Being | 50.9 (9.99) | 49.3 (9.78) | 47.9 (10.8) |
| -Parent Relation & Home Life | 54.6 (8.92) | 54.3 (9.87) | 48.7 (9.62) |
| -Social Support & Peers | 52.7 (9.22) | 51.8 (10.3) | 49.6 (11.8) |
| -School Environment | 52.3 (8.64) | 51.4 (9.34) | 49.5 (10.6) |

**Table 5**: Mean values and standard deviations, **11– to 17-year-olds**, n = 1306
